# Low-Resistance Membrane vs. High-Resistance Membrane Performance Utilizing Electrodialysis–Evaporator Hybrid System in Treating Reject Brine from Kuwait Desalination Plants

**DOI:** 10.3390/membranes14080163

**Published:** 2024-07-24

**Authors:** Bader S. Al-Anzi, Maryam K. Awadh

**Affiliations:** Environmental Sciences, College of Life Sciences, Kuwait University, P.O. Box 5969, Safat 13060, Kuwait; maryam.awadh@ku.edu.kw

**Keywords:** desalination, electrodialysis, current density, flowrate, salt production, cation exchange membrane, anion exchange membrane

## Abstract

This work is an effort to mitigate the existing environmental issues caused by brine discharge from Kuwait’s desalination plants and to find an economical and efficient way of managing reject brine from local desalination plants. Low- and high-resistance membranes (LRMs and HRMs, respectively) were used to produce salt and low-salinity water from brine effluent utilizing an electrodialysis (ED)–evaporator hybrid system. The effect of high current densities of 300, 400, and 500 A/m^2^ and brine flowrates of 450 and 500 L/h on the quality of produced salt and diluate were investigated for LRM and HRM. The recovered salt purity for LRM is up to 90.58%. Results show that the low-resistance membrane (LRM) achieved higher water recovery, energy consumption, desalination rate, operation time and ion removal rate than those of the high-resistance membrane (HRM) under the same operating conditions. The difference in concentration for 300 A/m^2^ between LRM and HRM increased from 0.93% at 10 min to 8.28% at 140 min. The difference in diluate concentration effluent is negligible for both membranes, whereas LRM produced higher concentrate effluent than HRM for all current densities and low flowrate (400 L/h). The maximum difference between LRM and HRM (with LRM achieving higher concentrations) is 10.7% for 400 A/m^2^. The permselectivity of LRM for monovalent cations decreased with current density, whereas the effect on permselectivity for HRM was insignificant for the current density values. The addition of a neutral cell was effective in reducing the buildup of divalent ions on the inner membrane of the cathode side.

## 1. Introduction

With the continuous expansion of the global population, the pollution of existing freshwater supplies, and limited freshwater resources, water scarcity has become a worldwide concern [1]. In the next 50 years, human population is expected to grow by 40–50%, which will put a tremendous strain on the earth’s limited freshwater resources [1]. To overcome such water shortages and challenges, almost all countries have employed seawater desalination to replenish their water resources to reduce the deficit between demand and supply. Around 86.55 million m^3^/day of potable freshwater is currently produced by desalination technologies worldwide [2]. About 48% of desalination plants are located in the Middle East and North Africa (MENA) region. Of that, Kuwait accounts for about 3.7% of the desalination capacity [3]. The desalination facilities in Kuwait include seven multi-stage flash distillation (MSF) plants with a capacity of 1.47 million m^3^/day, two reverse osmosis (RO) plants with a capacity of 0.17 million m^3^/day, and one recent multi-effect desalination plant (MED) with a capacity of 0.486 million m^3^/day [4]. All types of desalination plants produce highly concentrated waste effluent known as “reject brine” which negatively impacts the marine environment when returned back to the ocean without proper treatment. For example, the continuous discharge of brine to the marine system has been associated with serious localized negative impacts like eutrophication, elevated heavy metals, osmotic balance disruption, thermal pollution, and pH fluctuations, which highly affect the marine life fauna and flora [5,6]. Around 142 million m^3^ of brine is disposed of every day from desalination plants worldwide, i.e., around 1.5 times more than the world desalination capacity [7]. Therefore, the management of brine effluents from desalination plants is a significant challenge that needs to be addressed properly [7]. In general, desalination plants in Kuwait produce reject brine with a total dissolved solid (TDS) concentration of 70,000 ppm (i.e., almost twice the TDS of seawater) [8]. These rejects also contain various other contaminants that require proper disposal or treatment [1,9]. Many studies have explored approaches for the appropriate utilization of highly concentrated brine to mitigate its negative impact on the environment and to recover valuable elements [7,8,9]. These include the production of reusable solid products (sodium bicarbonate), the recovery of salt and minerals for commercial purposes, and the recovery of metals (magnesium (Mg), potassium (K), lithium (Li), palladium (Pd), radium (Ra), uranium (U), and scandium (Sc)) [10,11]. Other brine management applications include recovering energy from the indirect mixing of brine with low-salinity treated wastewater effluent utilizing Pressure-Retarded Osmosis (PRO) [12,13].

Membrane-based technologies have been shown to be a promising approach to recover minerals and produce low salinity water and wet/dry salt from concentrate rejects. Electrodialysis (ED) is one of the most cost-effective, feasible, and environmentally friendly technologies to recover solid salts from RO rejects [14,15,16,17]. Casas et al. [16] showed ED as a feasible technology in concentrating brine up to 280 g/L (NaCl), which can be an alternative source for the chlor-alkali industry. Tanaka et al. [17] proved that salt production from RO brine was more profitable than seawater and it saved 20% of energy. Pereira et al. [18] also reported that ED uses less energy than the evaporation process in concentrating brine. In Japan, an ED–evaporator hybrid system was used on a large scale to recover NaCl from seawater [17]. In an integrated RO-ED–crystallizer method, Nayar et al. [19] used RO brine instead of seawater in the ED diluate stream and found that it decreased water production cost by 87% while increased salt production cost by 26%. Jiang et al. [20] treated synthetic RO brine with a three-stage semi-batch ED system and reported a TDS final concentration of 271.3 g/L and a water recovery of 67.78%. Similar results were also reported by Yan et al. [21] that the ED system was successful in concentrating brine from 3.5 g/L to 20.6 g/L using a three-stage batch operation. Water transport increased with stage number from 17.8% to 42.5% in those studies, and the third stage was more energy-intensive than the other two stages. 

Integrating ED with bipolar membranes (EDM) could recover acid, base, and hypochlorite from the RO concentrate [22]. A previous study suggested that a combination of membrane and thermal-based technologies could lead to zero liquid discharge (ZLD) [1]. Nayar et al. [23] proposed a theoretical RO-ED–crystallizer technology to achieve zero brine discharge in seawater desalination. Compared to the standalone ED systems, the RO-ED system reduced brine concentration costs by 33–70%. Electrodialysis reversal (EDR) works similar to ED except that the direct current (DC) voltage is reversed 3–4 times every hour. Medina et al. [24] obtained a 92% water recovery using EDR operation in treating RO brine concentrate. He et al. [25] used a pre-treatment operation prior to the EDR process and achieved a 96% water recovery for the brine concentrate. ED technology was also used by Korngold et al. [26] and Korngold et al. [27] to reduce the volume of RO brine effluent and its disposal cost. 

Membrane fouling is a major challenge for all membrane technologies and chemical fouling is primarily caused by the precipitation of insoluble salt formed near the membrane. Scaling can be reduced by passing the brine through a separate CaSO_4_ precipitation tank containing gypsum seeds. The performance of a lab-scale hybrid system consisting of a pellet reactor and ED was evaluated by Tran et al. [28], and the results showed that the removal efficiency of calcium was between 70 and 80% and thus ED was operated in a stable condition with higher current efficiency without scaling. Zhang et al. [29] also studied the feasibility of ED in desalinating RO effluents in lab-scale and pilot-scale plants and found that 75% salt removal was achieved when operated in continuous mode. They concluded that the total operational cost of ED can be further reduced by subjecting the feedwater to a decarbonisation operation to reduce the scaling potential. Yan et al. [21] reported that the ED operation, especially water and salt transport and energy consumption, is heavily affected by the operating parameters (current density, feedwater flowrate, composition, temperature, and membrane stack size). Among many operational factors, the two most impactful factors on the ED performance are the feed flowrate and applied voltage [30]. Choi et al. [31] reported that ED exhibits properties suitable for the desalination of high-salinity solutions since feed solution with high conductance makes the process more energetically favourable. In our previous study [32], we studied the feasibility of operating ED in a multi-stage batch process using membranes with higher areal resistances (~20 Ωcm^2^). The effect of various high current densities and feed flowrates were also studied, and it was found that higher flowrate operation reduced the energy consumption, operation time, and system resistance of the process but it also reduced the ion removal rate. A recent study by Zhang et al. [33] suggested that the acidification of the diluate and concentrate stream of ED can further improve the efficiency of ED. In spite of this progress, however, several questions remain to be addressed such as the extent of the scaling potential effect, the feasibility of using a lower resistance membrane (LRM), the permselectivity of the membrane, and the effect of an additional neutral cell on the performance of the system. Permeselectivity is a measurement used to determine the membrane’s ability to measure the selectivity of ions while rejecting the passage of specific ions [34].

The aim of the current study is to characterize the LRM and HRM of the ED system used in the current study. Membrane characterization was achieved through investigating the permselectivity of LRM and HRM. The scaling potential due to calcium carbonates using the Langelier Saturation Index (LSI) was also investigated. LRM performance was assessed by investigating the effects of different operating conditions on water retention, desalination rate, energy consumption, operation time, and ion removal rate using monovalent membranes of lower resistances (5–6 Ωcm^2^). Furthermore, a neutral cell compartment was added near the cathode side, as per our previous study’s recommendation, in an attempt to reduce the divalent fouling formed on the end of the membranes. Finally, the performance of the lower resistance membrane (LRM) was compared to that of the higher resistance membrane (HRM) under the same operating conditions.

## 2. Materials and Methods

### 2.1. Materials 

Synthetic reject brine (feed water) was prepared by adding commercial-grade NaCl (97–99% purity) into tap water. The final concentration of the synthetic brine was around 65,000–70,000 ppm to mimic reject brine from Kuwait desalination plants [6]. The compositions of synthetic brine solution and tap water are given in Table 1. There was no addition of any other monovalent (e.g., K^+^) or divalent ions to the synthetic reject brine; the small concentration of divalent ions comes from the tap water and added salt. The data were evaluated through a software provided by PCell (PC Fronted Software, Version 1.4, PCell, GmbH, Heusweiler, Germany).

### 2.2. Electrodialysis Cell, Equipment Set Up, and Operation Procedure

**Figure 1:**
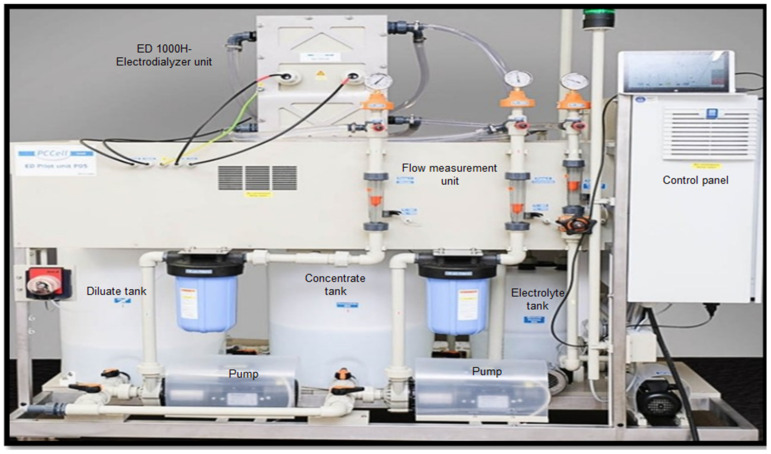
ED pilot unit used in the experiment with its main components marked.

**Figure 2:**
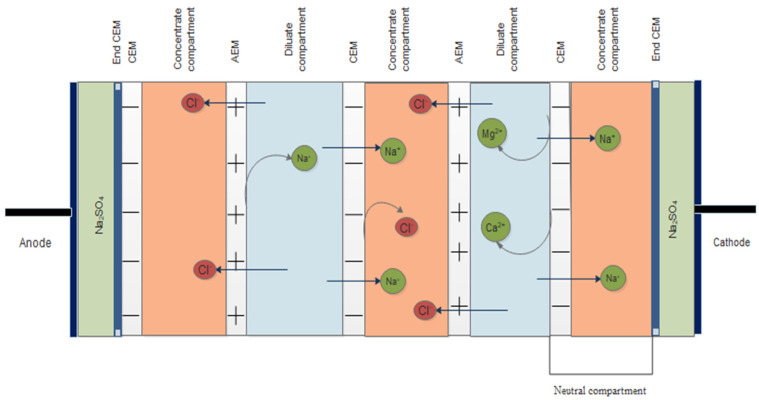
Schematic of the ED stack showing the neutral cell compartment near the cathode side.

Figure 1 shows the various components of the ED unit used in this study. The electrodialyzer unit is PC Cell ED 1000H (PC Cell GmbH, Heusweiler, Germany). This unit comprises a pre-assembled membrane pack with 25 cell pairs including one neutral cell on the cathode side with an active membrane area of 1000 cm^2^ for each membrane (Figure 2). Each cell pair consists of one monovalent cation and anion exchange membrane (PC-MVK and PC-MVA) and two spacers (diluate and concentrate). Spacers are made of polypropylene and have a thickness of 0.04 cm each. The characteristics of the membranes are given in Table 2 and Table 3. The equipment set up consists of 3 separate tanks with a capacity of 70 L for concentrate and diluate solutions, and 35 L for electrolyte solutions. A sodium sulphate solution of 60 ppm was used as the electrolyte solution throughout the experiment. The ED experiments were carried out in a one-stage-batch mode by filling the concentrate and diluate compartments initially with synthetic brine solution. The operating time for the ED operation was 140 min. The concentrate effluent from each batch was evaporated using a rotary evaporator (BUCHI Rotavapor R-200, Buchi AG, Flawil, Switzerland) to produce coarse salt as the final product, and the purity of the obtained salts was later compared with each other in this study. Different flowrates (Q) (400 and 450 L/h) and current densities (I_d_) (300, 400, and 500 A/m^2^) were used in the current experiments to determine their effects on the operating time, energy consumption, current efficiency, ion removal rate, desalination rate, and water recovery rate for LRM. In view of the challenges encountered during our previous study due to the accumulation of divalent ions (Ca^2+^ and Mg^2+^) on the end cation exchange membrane (end CEM) near the cathode side, a neutral compartment consisting of an additional cation exchange membrane (CEM) and a concentrate spacer was installed at the end CEM near the cathode side to reduce fouling on the end CEM. The extent of fouling was analyzed using a scanning electron microscope (SEM, JSM-6010LA, Jeol, Tokyo, Japan). 

### 2.3. Sampling and Analytical Methods

Samples from the diluate and concentrate tanks were collected at the end of each run. The concentration of cations including Na^+^, K^+^, Mg^2+^, and Ca^2+^ were measured using a microwave plasma-atomic emission spectrometer (MP-AES, Agilent: 4200, Mulgrave, VIC, Australia), and the contents of the anions were analyzed using ion chromatography–mass spectrometry (IC-MS, Metrohm 850, Metrohm, Ionenstrasse, Switzerland). The current, flowrate, specific conductance, and pH were measured on-site with the pilot-scale instrument.

### 2.4. Data Analysis and Calculation

#### 2.4.1. Langelier Saturation Index (LSI)

LSI indicates the degree of saturation of calcium carbonate in water. It is calculated using the actual *p*H (measured) and the saturation value *p*H_s_ of the water, according to Equation (1) [28,33]

(1) $$LSI=pH-pH_{s}$$

$$pH_{s}=\left(9.3+A+B\right)-(C-D)$$

where

$$A={(log}_{10}\left[TDS\right]-1)/10$$

$$B=-13.12\times {log}_{10}\left[T\right]+134.55\;$$

$$C={log}_{10}[{\mathrm{Ca}}^{2+}]-0.4$$

$$D={log}_{10}[alk]$$

[TDS]: The concentration of total dissolved solids in the solution measured in (mg/L).

[T]: Temperature in Kelvin (K).

[Ca^2+^]: The concentration of calcium as CaCO_3_ in (mg/L) in the solution.

[*alk*]: The concentration of alkalinity of CaCO_3_ in (mg/L) in the solution. 

#### 2.4.2. Desalination Rate (D)

The desalination rate was calculated using Equation (2) [14]

(2) $$D=\frac{S_{d,0-}S_{d,t}}{S_{d,0}}\times 100\%$$

where S_d,t_ and S_d,0_ are the conductivities (µs/cm) of diluate tank at time t and 0.

#### 2.4.3. Ion Removal Rate (R)

The rate at which ions were removed from the diluate tank was calculated using Equation (3) [32]

(3) $$R=\frac{C_{d,i}-C_{d,f}}{C_{d,i}}\times 100\%$$

where $C_{d,i}$ and $C_{d,f}$ are the initial and final concentrations of the ions in the diluate tank (ppm).

#### 2.4.4. ED Membrane Permselectivity 

The permselectivity of monovalent ions to divalent ions ($P_{div}^{monov})$ was calculated using Equation (4), using the experimental results obtained by measuring the change in the relative concentration of monovalent ions to divalent ions [35]

(4) $$P_{div}^{monov}=\frac{C_{monov,d,i}-C_{monov,d,f}}{C_{monov,d,i}}/\frac{C_{div,d,i}-C_{div,d,f}}{C_{div,d,i}}$$

where $C_{monov,d,i}$ is the initial concentration of monovalent ions in the diluate, $C_{monov,d,f}$ is the final concentration value of monovalent ions in the diluate, $C_{div,d,i}$ is the initial concentration of divalent ions in the diluate, and $C_{div,d,f}$ is the final concentration of divalent ions in the diluate. 

#### 2.4.5. Water Recovery (W) 

The water recovery rate was calculated using Equation (5) [14,20]

(5) $$W=\frac{V_{d,f}}{V_{d,i}}\times 100\%$$

where V_d,i_ and V_d,f_ are the initial and final volumes of the diluate tank (L). 

#### 2.4.6. Current Efficiency (CE) 

The current efficiency was calculated using Equation (6) [14]

(6) $$CE=\frac{zFV_{t}(C_{t}-C_{0})}{NIt}\times 100\%$$

where C_0_ and C_t_ are the initial and final concentrations of Na^+^ in the concentrate tank (mol/L), z is the ion charge, F is the Faraday number (96,485 A s/mol), V_t_ is the volume of the concentrate tank (L) at time *t*, N is the number of cell pairs, I is the current (A), and *t* is the operation time (s). 

#### 2.4.7. Energy Consumption (E)

The energy consumption for the desalination process was calculated using Equation (7) [32]

(7) $$E=\frac{I\times U\times t}{1000}$$

where E is the energy consumption, I is the current (A), U is the voltage (V), and t is the operation time (h). 

#### 2.4.8. Purity of Salt (P) 

The purity of the final coarse salt was determined by the following Equation (8) [14]

(8) $$P=\frac{C_{f}V_{f}M_{b}}{W_{f}}\times 100$$

where C_f_ is the final concentration of Na^+^ in the concentrate tank (g/L), V_f_ the final volume of the concentrate compartment (L), M_b_ is the molecular weight of NaCl (g/mol), and W_f_ is the final dry solid weight of the coarse salt recovered after evaporation (g).

## 3. Results and Discussion

### 3.1. Permselectivity of LRM vs. HRM

#### 3.1.1. LRM and HRM Cation Monovalent Selectivity

**Figure 3:**
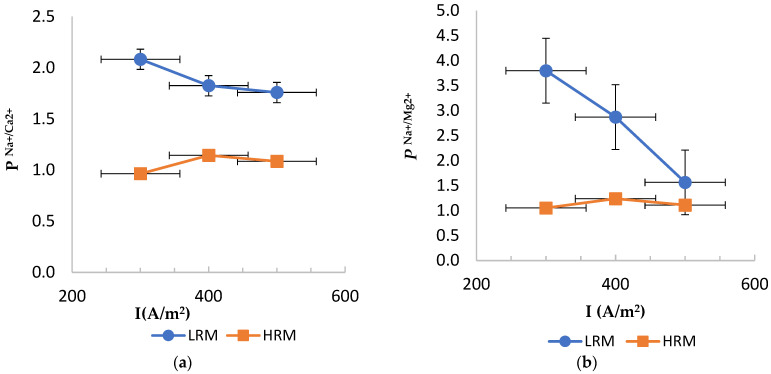
Permselectivity of LRM and HRM at various current densities for (**a**) $P_{{Ca}^{2+}}^{{Na}^{+}}$ and (**b**) $P_{{Mg}^{2+}}^{{Na}^{+}}.$

Before discussing the results, one should mention that during the preparation of the synthetic brine, no divalent ions were added to the solution. The number of divalent ions found in the solution is from the tap water and a small impurity in the added salt; however, it is negligible. The percentage of divalent ions in the synthetic brine were 0.017% and 0.062% for Mg^2+^ and Ca^2+^, respectively, compared to the added NaCl solution value of 31.7% and 54.21% for Na^+^ and Cl^−^, respectively. Figure 3a,b compares the permselectivity of LRMs and HRMs for $P_{{Ca}^{2+}}^{{Na}^{+}}$ and $P_{{Mg}^{2+}}^{{Na}^{+}}$ at various current densities and a flowrate of 400 L/h. While HRM was not cation monovalent-selective, LRM showed cation monovalent selectivity that decreased with current density. LRM was more selective in retaining magnesium ions than calcium ions. At 300 A/m^2^, the selectivity to sodium was more than two-fold that of calcium ions and nearly four-fold that of magnesium ions. At high current density (500 A/m^2^), selectivity reduced, with sodium selectivity only ~1.75 times that of calcium and ~1.5 times that of magnesium. Previous studies reported that low current density can improve the efficiency of the monovalent selective membranes to separate monovalent and divalent ions [14], which agrees with the current findings.

#### 3.1.2. *Overall Salinity Profile*

**Figure 4:**
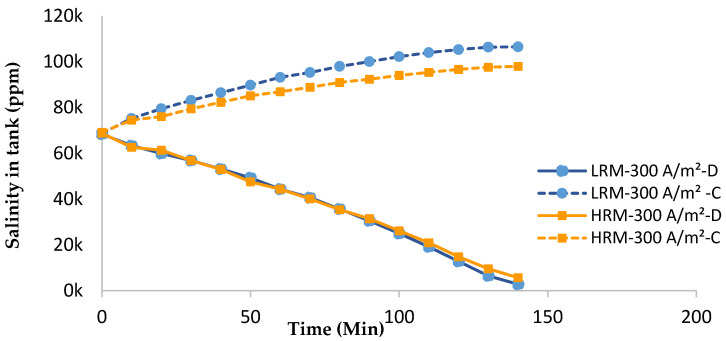
Concentration changes of changes in LRMs and HRMs at 300 A/m^2^ current density and a flowrate of 400 L/h.

The overall salinity profile of the diluate (D) and concentrate (C) of LRMs and HRMs at a current density of 300 A/m^2^ and a flowrate of 400 is shown in Figure 4. While the overall salinity profile of the LRM and HRM diluate streams were similar, LRMs were able to achieve a noticeably higher overall concentration (that increases time) than HRMs, indicating that LRMs were less permeable to water transport [32].

#### 3.1.3. Effect of Monovalent Selectivity of LRM and HRM on Cations in the Diluate

**Figure 5:**
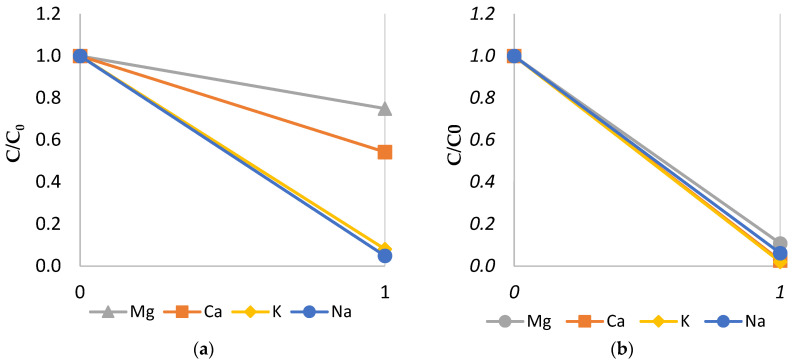
Normalized concentration changes for 300 A/m^2^ between initial and final states of cation concentration in diluate of (**a**) LRMs and (**b**) HRMs.

The impact of the monovalent selectivity of LRM and HRM on the relative concentration of sodium, potassium, calcium, and magnesium in the diluate can be seen in Figure 5a,b, respectively, with the x-axis representing the initial and final states in the diluate concentrations and the y-axis representing the normalized concentration with a concentration set up at the beginning of testing. The concentrations shown were for the case of a flowrate of 400 L/h and a current density of 300 A/m^2^. The LRM shown in Figure 5a was selective to monovalent cations and removed them more than the divalent ions. While sodium and potassium concentrations were reduced by 95% and 92%, respectively, the calcium and magnesium concentrations were only reduced by 46% and 25%. However, it is important to note that the initial concentration of the divalent ions (Ca^2+^ and Mg^2+^) were significantly lower than that of the monovalent ions (Na^+^). Figure 5b shows the relative concentration of the initial and final states of the HRMs, which were not selective to monovalent ions. 

### 3.2. Performance of LRM and HRM under the Same Operating Conditions

#### 3.2.1. Influence of Current Densities and Feed Flowrates

**Figure 6:**
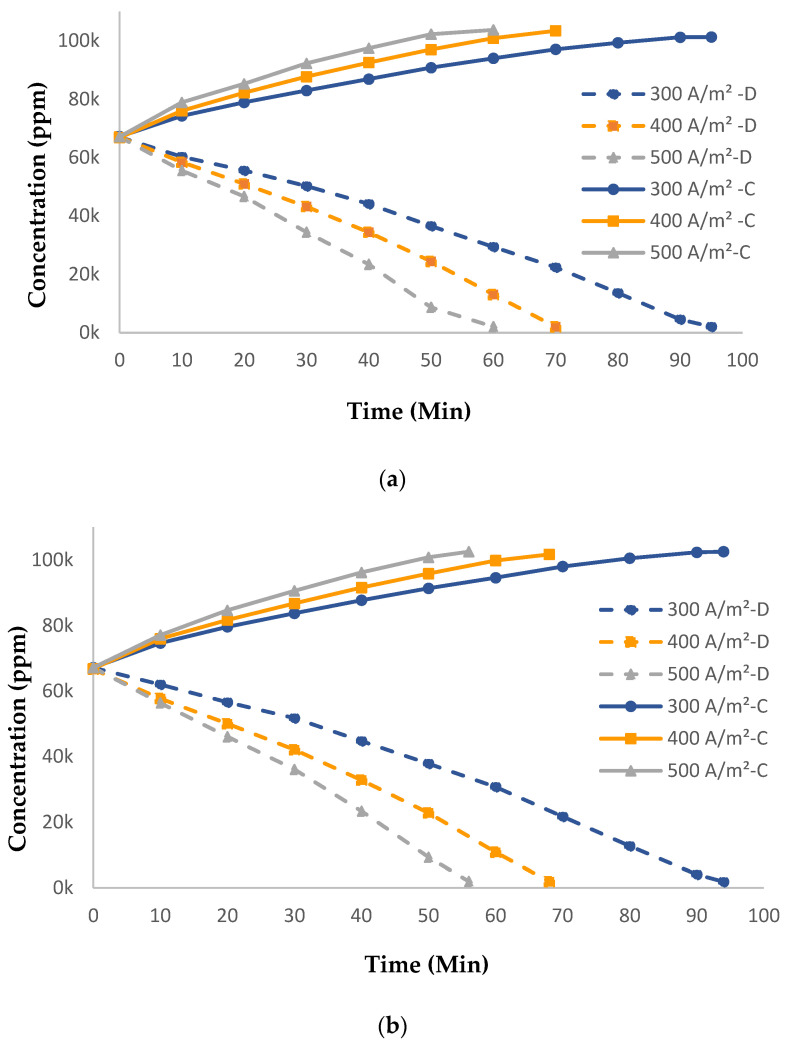
Change in the concentration of solution with different current densities at (**a**) 400 L/h and (**b**) 450 L/h the LRMs.

Figure 6a,b demonstrate how the concentration of salt in the concentrate tank (C) and diluate tank (D) changed with different current densities and flowrates. Higher current density greatly reduced the time needed to achieve the maximum concentration in the concentrate tank. When the applied current was raised from 300 A/m^2^ to 500 A/m^2^ (66.6%), the operation time was decreased by 35 min (36.8%) while maintaining a high desalination rate of 97% in the diluate tank for all experimental runs. This is due to the fact that at higher current densities, the ion-migration flux through the same membranes is faster [20,29]. In addition to shortening the operation time, higher current density (500 A/m^2^) resulted in a slight increase (2.36%) in the final salt concentration in the concentrate tank. With an increase in the feed flowrate by 12.5% (from 400 L/h to 450 L/h), the reduction in operation time was 8% while maintaining the same final concentration in the concentrate tank regardless of the applied current. This may be due to the decrease in the residence time of ions inside the ED stack at higher flowrates [36].

#### 3.2.2. Water Recovery

**Figure 7:**
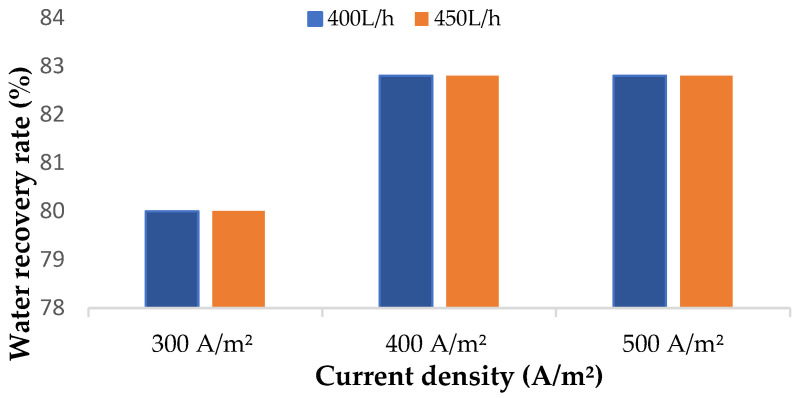
Water recovery rates applied at different current densities and feed flowrates.

The fundamental properties of ED membranes are to selectively transport ions and minimize water transport (maximum water recovery) [37]. Water transport in ion exchange membranes can happen because of osmosis (due to the concentration gradient of solution between the membranes) and electro-osmosis (due to the difference in electrical potential) [15,38]. The latter phenomenon is more evident in studies where high current densities are applied [32]. According to Galama et al. [38] water transport from dilute tank is more effective at lower current densities. This was evident in this study, where the amount of water recovered in the dilute tank increased as the density of the current increased (Figure 7). On the other hand, the increase in feed flowrate resulted in a slight increase in water transport with no impact on the water recovery, regardless of the current density. The concurrent transport of water molecules with ions across the membranes at higher current densities can be explained by the differences in concentration in the concentrate tank after 60 min of desalination at 300 A/m^2^ and 500 A/m^2^. Moreover, it is crucial to have minimum water transfer to the concentrate compartment when salt production from the concentrate compartment is to be achieved [20].

#### 3.2.3. Energy Consumption 

**Figure 8:**
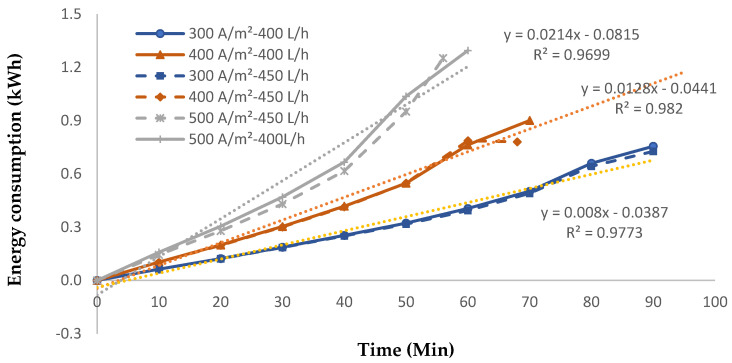
Energy consumption for 400 and 450 L/h at various current densities.

Figure 8 shows energy consumption changes over time for a range of current densities from 300 A/m^2^ to 500 A/m^2^ at two different flowrates (400 L/h and 450 L/h). For all current densities and feed flowrates, energy consumption increased linearly with operating time, with R^2^ for all current densities and flowrates being greater than 0.97. Based on Equation (7), higher current densities lead to a rise in energy consumption. This is shown by the slope of the fitted model, where the slope of 500 A/m^2^ data is 1.8 and 2.7 folds that of 400 A/m^2^ and 300 A/m^2^, respectively. 

**Figure 9:**
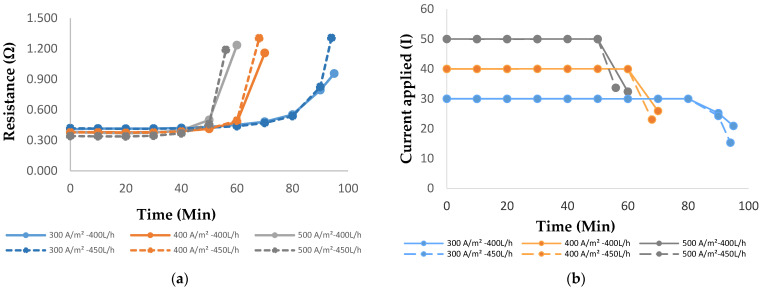
(**a**) System resistance vs. time, and (**b**) current applied vs. time for both flowrates.

Figure 9a shows a sharp rise in the resistance toward the end of the run for all current densities and flowrates. This is due to salt removal from the highly diluted diluate compartment that increased the applied voltage during desalination for both flowrates. The depletion of salt in the diluate compartment (which could be observed when the desalination rate was between 80 and 87%) resulted in a rise in the voltage of the system and thereby the energy consumption [14,25]. The reason for the significant rise in system resistance even during the use of membranes with low areal resistance (5 and 6 Ω.cm^2^) can be attributed to the dominance of solution resistance at the low salt concentration (which can be observed in Figure 9a,b with the sharp increase in the resistance and decrease in current, respectively) [32].

### 3.3. Performance Comparison between HRM and LRM 

**Figure 10:**
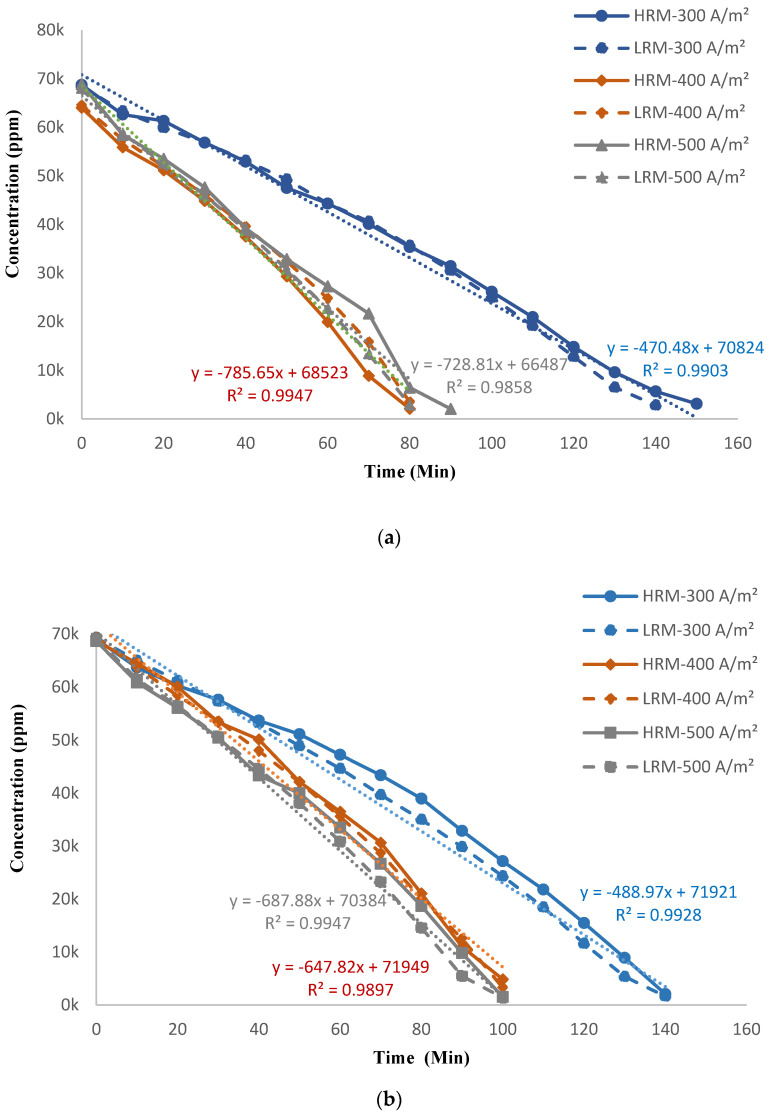

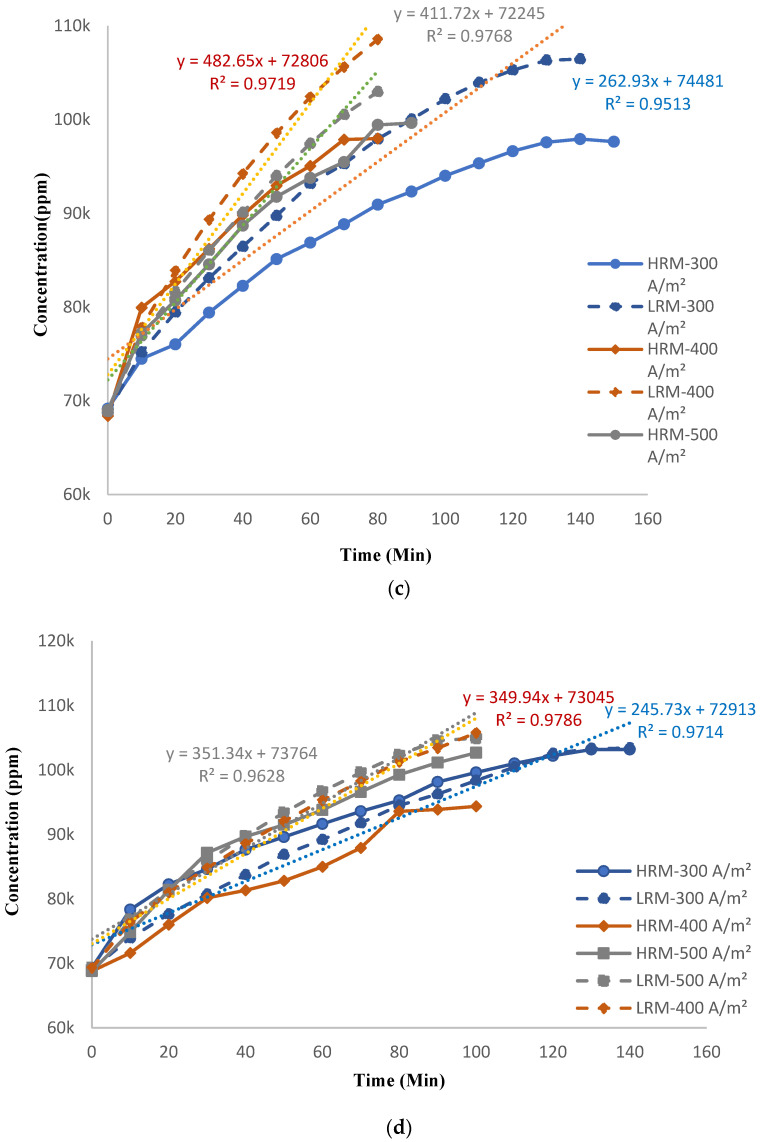
Change in the concentrations obtained using HRM and LRM for different current densities at a flowrate of (**a**) 400 L/h in the diluate tank, (**b**) 450 L/h in the diluate tank, (**c**) 400 L/h in the concentrate tank, and (**d**) 450 L/h in the concentrate tank.

Figure 10 shows the change in the concentration of diluate and concentrate tanks for different current densities at 400 and 450 L/h flowrates utilizing HRM and LRM. In spite of the fact that the initial feed concentration of HRM is higher than that of LRM by 420 ppm, the final salt concentration obtained in the concentrate tank using LRM was higher at all current densities (I_d_) and flowrates (Q) in comparison with that of HRM (Figure 10c,d). Figure 10a,b show that the diluate concentration for all current densities and flowrates decreased linearly with the slope ranging from −470 to −785 (steeper slope at higher I_d_) and an R^2^ of 0.99 for both HRM and LRM. The difference between HRM and LRM throughout the run was negligible. A similar linear trend was also observed for the concentrate concentration with an R^2^ between 0.95 and 0.97 for both membranes. It has been noticed that linearity improves with current density. The difference between HRM and LRM increased with time in all scenarios (Figure 10c,d). For example, the difference in concentration for 300 A/m^2^ between LRM and HRM increased from 0.93% at 10 min to 8.28% at 140 min (Figure 10c). The maximum difference between the membranes (with LRM achieving higher concentrations) for other current densities is 10.7% and 3.22% for 400 A/m^2^ and 500 A/m^2^, respectively. At a higher flowrate (450 L/h) the difference between LRM and HRM is small except for 400 A/m^2^. This may be due to the lower residence time of ions at high flowrate. The desalination rate using both membrane sets in the diluate tank was similar for both flowrates. Takagi et al. [39] suggested that the permeability/mobility of ions across the membranes is determined by membrane resistance. This resistance is not constant and is affected by both the external solution concentration and by membrane fabrication. The results from our study agree with this observation, as the membranes with low areal resistance showed higher ion permeability.

As shown in Table 4, the CE of the process improved from 30.8% to 52.3% for 300 A/m^2^ for the same flowrate, 400 L/h. This applies to all current densities and flowrates when using LRM rather than HRM. As indicated by Equation (6), CE is directly proportional to the ΔC of Na^+^ and V_t_. Even though the final concentrate tank volume using HRM is higher than that of LRM (by 420 ppm), the ΔC term of LRM overcomes the V_t_ term of HRM that results in a higher CE using LRM. Other variables in Equation (6) were almost similar for both studies. The lower CE value of HRM can be attributed to the lower Na^+^ permeability through the membrane. The concentration polarization effect is negligible in this study because it is a one-stage experiment in which the final salt concentration is not as high as in our previous study (multi-stage) [32]. In addition, since higher current densities are used, the effect of back-diffusion is also negligible. Furthermore, Tedesco et al. [40] stated in their study that using low-resistance membranes and an ED stack with a smaller compartment thickness is critical for improving process efficiency. The spacer used in this study has a thickness of 0.4 mm, which is less than the thickness of the spacer used in the HRM-ED stack. As a result of the thinner spacer, the overall compartment thickness is reduced. This could also be a contributing factor for better overall performance using LRM.

The ion removal rate was investigated for LRM and HRM under the same operating conditions. Zhang et al. [14] stated that a low current density can improve the efficiency of monovalent-selective membranes to separate mono- and divalent ions. Table 5 shows that the divalent ion removal rate for LRM is significantly lower than that of LRM, especially at a low flowrate. This confirms that LRM is a monovalent-selective membrane, as stated previously in this study. The reduction in the transportation of divalent ions improves the efficiency of the process as well as the purity of the salt produced. Results show that as the flowrate increases, there is an increase in the removal of ions to the concentrate tank. This increase in the removal rate of ions can be due to the thinning of the interface between the membrane surface and the adjacent liquid at higher flowrates.

### 3.4. Scale Formation and Prevention 

#### 3.4.1. Langlier Saturation Index (LSI) of the Feed Water

The LSI of the feed water was −0.866. According to a previous study [32], LSI in the range of −1 to +1 indicates that the water is relatively not corrosive to metallic components of the distribution system [41]. Therefore, no additional pH adjustment by acidification (using HCl) for decarbonization was performed in this study.

#### 3.4.2. Assessment of the Addition of the Neural Cell to the ED System

**Figure 11:**
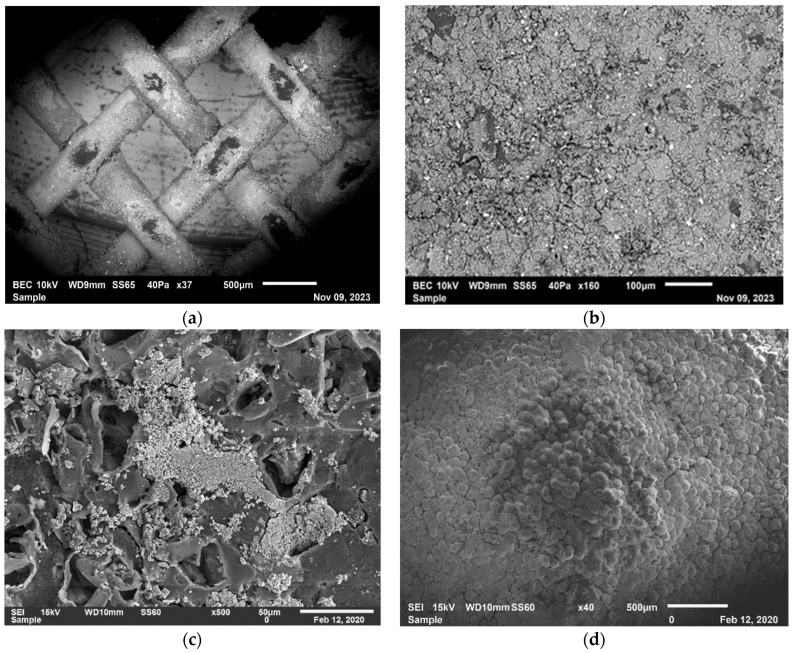
Images of (**a**) fouled-up neutral cell incorporated in LRM. (**b**) CEM near the cathode side incorporated in LRM. (**c**,**d**) illustrate the effect of scaling in the HRM, a fouled-up end CEM [32].

**Figure 12:**
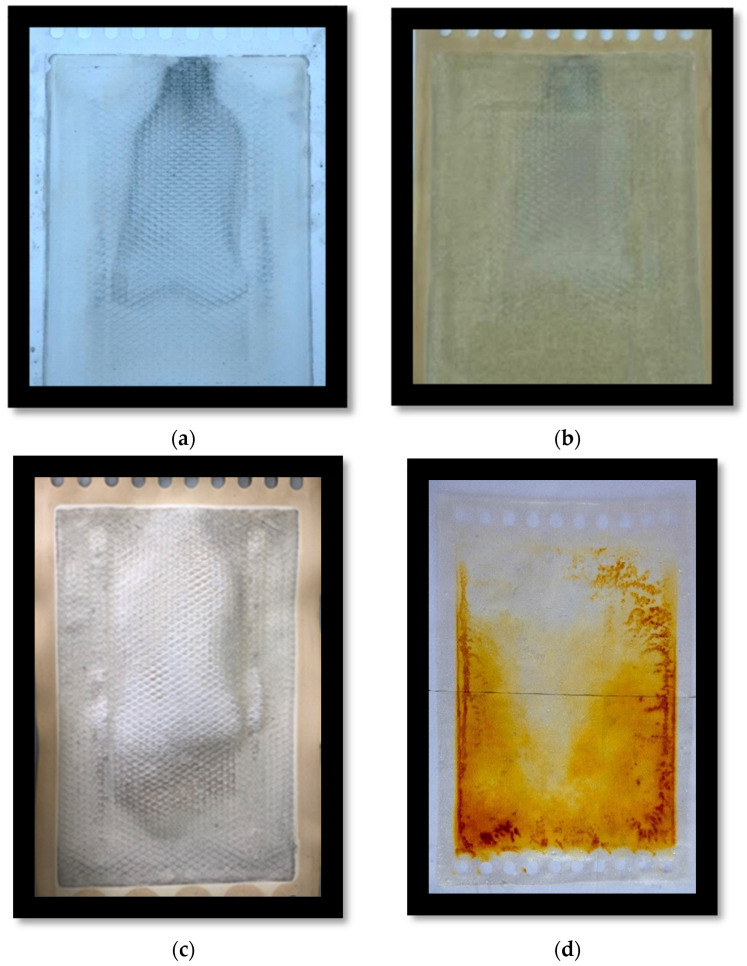
(**a**) Fouled-up neutral cell in LRM, (**b**) slight fouling in CEM in LRM (**c**) fouled-up CEM in HRM, and (**d**) high fouling on CEM in HRM.

It was suggested in our previous study [32] to assess the impact of adding a “neutral cell” as a 25th cell on the performance ED system. A neutral cell contains an additional END-CEM layer added to the ED stack in the current study before the inner cation exchange membrane near the cathode side. The additional layer was added in an attempt to reduce the scale formation accumulated on the cathode side caused by the divalent ions (Mg^+2^ and Ca^+2^). These cations are influenced by cathode attraction migrated to the diluate spacer near the cathode side causing scaling [32]. CEM is known to be more expensive compared to the neutral cell, which shows the importance of protecting such membranes and the entire membrane stack of the ED system starting with the inner membrane of the cathode side. A neutral cell is cost-effective and can easily be replaced as needed. After conducting the experiment, the membrane was dissembled and was taken for further analysis using a SEM- EDX to conduct a comparative study with that of HRM for more details and information on how this cell impacted the performance of the ED system. The obtained results are shown in Figure 11 and Figure 12.

## 4. Conclusions

Since the LSI number indicated that the feed brine water was not corrosive, there was no need for pH adjustment. One of the important outcomes of this study is the characterization of LRM vs. HRM with regard to permselectivity. LRM is a monovalent-selective membrane as shown by the permselectivity study, with a lower removal rate of divalent ions than HRM. The ED operating time was shortened by increasing current densities and feed flowrates. The amount of water retained in the dilute tank (WRR) increased with current density. Solution resistance predominates especially at low salt concentration, causing significant energy consumption. The highest salt purity of 90.58% was observed for 500 A/m^2^ with 400 L/h. The concentration of the diluate and concentrate tanks behaved linearly with time for HRM and LRM. The difference in the concentration of the diluate tank for both membranes (HRM and LRM) was negligible; however, there was a clear difference in the concentrate tank between HRM and LRM that increased with time and decreased with flowrate. More WRR was achieved with LRM that increased with system feed flowrate and current density. The CE was significantly improved in the case of LRM for a current density of 300 A/m^2^ and a flowrate of 400 L/h. For purer salt in terms of monovalent ions (Na^+^ and Cl^−^), LRM is more suitable (since it is monovalent-selective); however, for lower water salinity in the diluate, HRM is more suitable (since it removes both monovalent and divalent ions). The extra neutral cell that was added acted as a sacrificial membrane to protect the more expensive CEM by reducing the formation of fouling on the CEM. 

## Figures and Tables

**Table 1:** Properties of synthetic brine solution (S) and tap water (T) used in this study.

Properties	Concentration (S)	Concentration (T)
Conductivity (µs/cm)	95,790	220
pH	7.5	10.2
TDS (ppm)	67,053	154
Alkalinity (ppm)	61	51
Sodium (ppm)	21,300	14.6
Magnesium (ppm)	11	6.5
Calcium (ppm)	42	1.9
Potassium (ppm)	120	1.3
Chloride (ppm)	36,354	18.1
Sulphate (ppm)	1866	8.9

**Table 2:** Electrodialysis stack parameters.

Parameter	Value
ED stack model	PC Cell ED 1000H
Number of cell pairs (*n*_cp_)	25
Active membrane area (*A*_m,active_)	1000 cm^2^
Channel height (*h*)	0.4 mm
Channel flow width (*w*_m,flow_)	27 cm
Capacity of diluate tank (*V*_d,cap_)	70 L
Capacity of concentrate tank (*V*_c,cap_)	70 L

**Table 3:** Characteristics of PC-MVA and PC-MVK membranes ^a,b^ [32].

	Membrane	Thickness (μm)	pH Stability	Area Resistance (Ω.cm^2^)	Burst Strength (kg.cm^−2^)	Max. Temperature (°C)	Reinforcement
LRM	PC-MVA	110	0–9	5	4–5	60	Polyester
	PC-MVK	100–120	0–10	6	4–5	45	Polyester
HRM	PC-MVA	110	0–9	20	2	60	PVC
	PC-MVK	100	0–10	-	3	40	PVC

^a^ The data were from the manufacturer. ^b^ PC-MVA and PC-MVK membranes were procured from PC Cell- GMbh along with the PC Cell P05 pilot ED unit.

**Table 4:** Comparison of stack performance parameters of operational time, WRR, stack energy consumption, and CE between LRM and HRM stacks at a current density of 300 A/m^2^ and a flowrate of 400 L/h.

Membrane Type	Current (A/m^2^)	Flowrate L/h	Volume (L)	Operational Time (min)	WRR (%)	EC (kwh)	CE (%)
LRM	300	400	50	80	79.17	0.546	52.30
HRM	300	400	50	150	70.83	1.15	30.80

**Table 5:** Ion removal rate for monovalent and divalent ions collected from the diluate tank for both HRMs and LRMs.

		HRM			LRM	
Ion	300 A/m^2^	400 A/m^2^	500 A/m^2^	300 A/m^2^	400 A/m^2^	500 A/m^2^
Ca^+2^	97%	86%	91%	45.7%	51%	53%
K^+^	98%	96%	90%	91.95%	92%	92%
Na^+^	94%	98%	98%	95%	92%	92%
Mg^+2^	89%	79%	89%	25.31%	32%	59%
Cl^−^	99.8%	95%	94%	95.70%	95.20%	95%

## Data Availability

Data are contained within the article and Supplementary Materials, further inquiries can be directed to the corresponding author.

## Supplementary Materials

The following supporting information can be downloaded at: https://www.mdpi.com/article/10.3390/membranes14080163/s1, Figure S1: Salts recovered from various operating conditions; Figure S2: ED Configuration; Figure S3: SEM images (a) Neutral Cell, (b) CEM in LRM, (c) CEM in HRM; Table S1: Glossary of all terms used in the study; Table S2: Salt Purity Values.
