# Supplementary material for: Low-Resistance Membrane vs. High-Resistance Membrane Performance Utilizing Electrodialysis–Evaporator Hybrid System in Treating Reject Brine from Kuwait Desalination Plants
Source: Membranes (Basel). 2024 Jul 24;14(8):163. doi: 10.3390/membranes14080163 (PMC11356419; doi:10.3390/membranes14080163)
Supplement: Supplementary file 1 [file membranes-14-00163-s001.zip › membranes-3067148-supplementary.pdf]

Supplementary Material:

Symbol	*Meaning*
LRM	Low -Resistance Membrane
HRM	High Resistance Membrane
RO	Reverse Osmosis
MED	Multi-effect desalination
TDS	Total Dissolved Solid
ED	Electrodialysis
ZlD	Zero-Liquid Discharge
EDR	Electrodialysis reversal
Id	Current Density
Q	Flow Rate
LSI	Langier Saturation Index
DR	Desalination Rate
R	Ion Removal Rate
P_b^a	Perm-Selectivity of (a / b)
WRR	Water Retention Rate
CE	Current Efficiency
E	Stack Electrical Energy Consumption
P	Purity of Salt
AEM	Anode Exchange Membrane
CEM	Cation Exchange Membrane

Supplementary Table S1: Glossary of all terms used in the study.

Sl.No.	Operating conditions	Salt recovered (%)	Purity of salt (%)	Sulphate (mg/Kg)	Magnesium (mg/Kg)	Sodium (mg/Kg)	Chloride (mg/Kg)	Potassium (mg/Kg)
**A**	300A/m^2-400L/h	97.81	89.58	2440	2008	409140	571150	8590
**B**	400A/m^2-400L/h	97.10	66.44	2950	1998	227340	599000	9090
**C**	500A/m^2-400L/h	97.10	90.58	1390	2008	404320	552330	13820
**D**	300A/m^2-450L/h	93.18	90.14	1310	2006	407150	564450	19250
**E**	400A/m^2-450L/h	96.26	86.18	2820	2004	406970	499490	9830
**F**	500A/m^2-450L/h	95.38	75.97	1050	2008	389200	551610	11390

Supplementary Table S2: Salt Purity Values

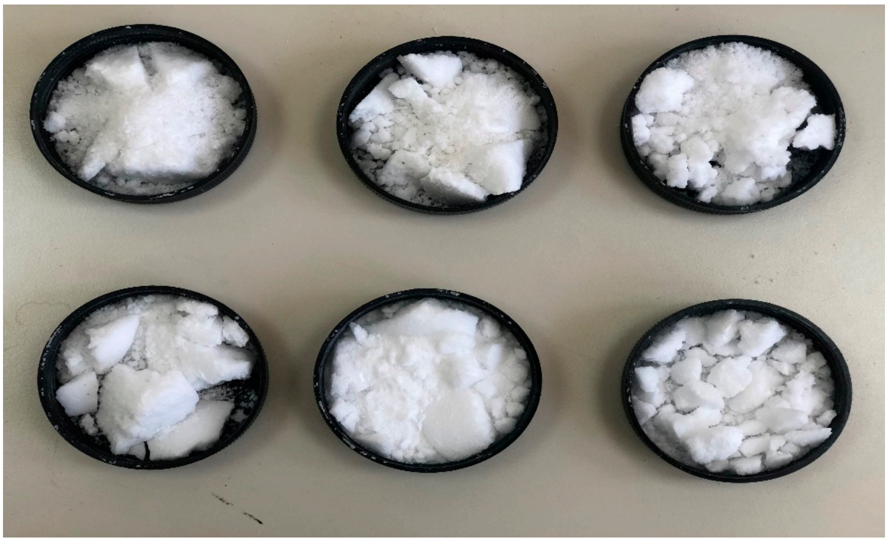

Supplementary Material Figure S1 . *Salts recovered from various operating conditions.*

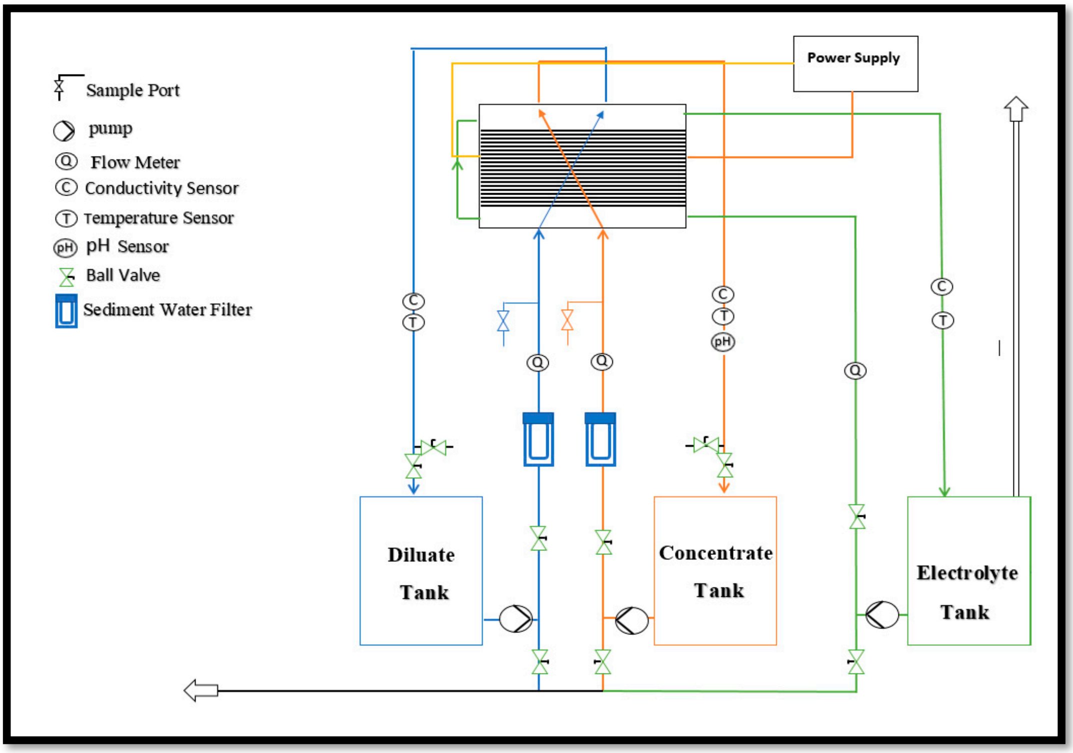

Supplementary Material Figure S2: ED Configuration

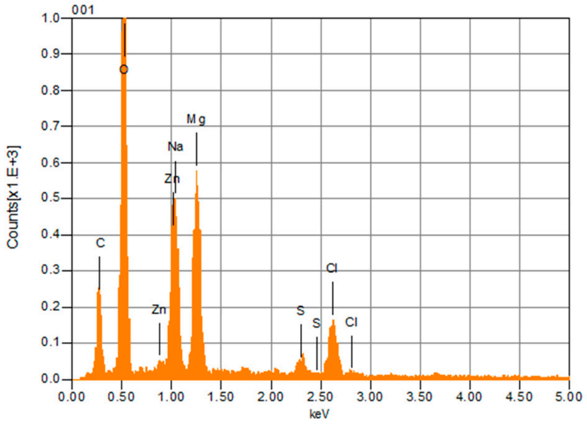

(a)

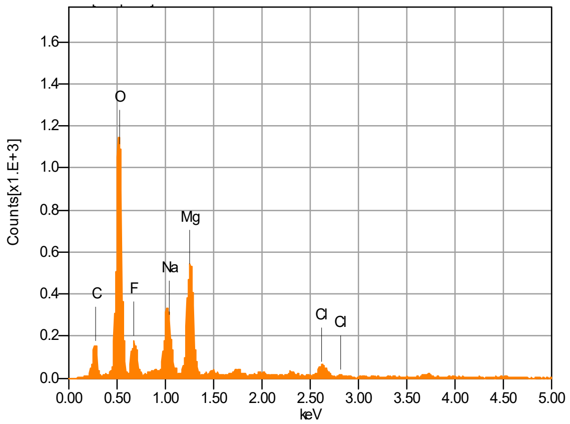

(b)

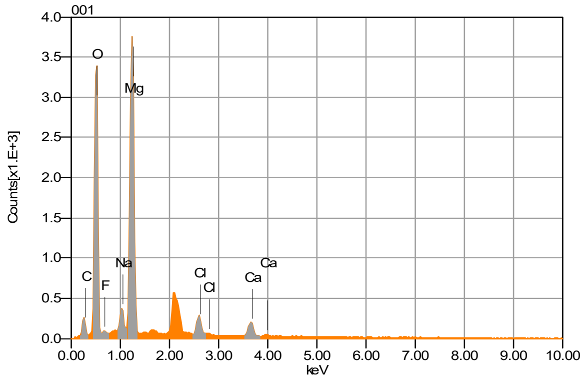

(c)

Supplementary Material Figure S3: SEM images (a) Neutral Cell, (b) CEM in LRM , (c) CEM in HRM
